# A Hybrid Approach of Using Wavelets and Fuzzy Clustering for Classifying Multispectral Florescence In Situ Hybridization Images

**DOI:** 10.1155/IJBI/2006/54532

**Published:** 2006-08-07

**Authors:** Yu-Ping Wang, Ashok Kumar Dandpat

**Affiliations:** Computer Science and Electrical Engineering Department, School of Computing and Engineering, University of Missouri-Kansas City, MO 64110, USA

## Abstract

Multicolor or multiplex fluorescence in situ hybridization (M-FISH) imaging is a recently developed molecular cytogenetic diagnosis technique for rapid visualization of genomic aberrations at the chromosomal level. By the simultaneous use of all 24 human chromosome painting probes, M-FISH imaging facilitates precise identification of complex chromosomal rearrangements that are responsible for cancers and genetic diseases. The current approaches, however, cannot have the precision sufficient for clinical use. The reliability of the technique depends primarily on the accurate pixel-wise classification, that is, assigning each pixel into one of the 24 classes of chromosomes based on its six-channel spectral representations. In the paper we introduce a novel approach to improve the accuracy of pixel-wise classification. The approach is based on the combination of fuzzy clustering and wavelet normalization. Two wavelet-based algorithms are used to reduce redundancies and to correct misalignments between multichannel FISH images. In comparison with conventional algorithms, the wavelet-based approaches offer more advantages such as the adaptive feature selection and accurate image registration. The algorithms have been tested on images from normal cells, showing the improvement in classification accuracy. The increased accuracy of pixel-wise classification will improve the reliability of the M-FISH imaging technique in identifying subtle and cryptic chromosomal abnormalities for cancer diagnosis and genetic disorder research.


Hindawi Publishing Corporation
International Journal of Biomedical Imaging
Volume 2006, Article ID 54532, Pages 1–11
DOI 10.1155/IJBI/2006/54532

### A Hybrid Approach of Using Wavelets and Fuzzy Clustering for Classifying Multispectral Florescence In Situ Hybridization Images

**Yu-Ping Wang and Ashok Kumar Dandpat**

*Computer Science and Electrical Engineering Department, School of Computing and Engineering, University of Missouri-Kansas City, MO 64110, USA*

Received 20 November 2005; Revised 21 April 2006; Accepted 25 April 2006

Recommended for Publication by Yue Wang

Multicolor or multiplex fluorescence in situ hybridization (M-FISH) imaging is a recently developed molecular cytogenetic diagnosis technique for rapid visualization of genomic aberrations at the chromosomal level. By the simultaneous use of all 24 human chromosome painting probes, M-FISH imaging facilitates precise identification of complex chromosomal rearrangements that are responsible for cancers and genetic diseases. The current approaches, however, cannot have the precision sufficient for clinical use. The reliability of the technique depends primarily on the accurate pixel-wise classification, that is, assigning each pixel into one of the 24 classes of chromosomes based on its six-channel spectral representations. In the paper we introduce a novel approach to improve the accuracy of pixel-wise classification. The approach is based on the combination of fuzzy clustering and wavelet normalization. Two wavelet-based algorithms are used to reduce redundancies and to correct misalignments between multichannel FISH images. In comparison with conventional algorithms, the wavelet-based approaches offer more advantages such as the adaptive feature selection and accurate image registration. The algorithms have been tested on images from normal cells, showing the improvement in classification accuracy. The increased accuracy of pixel-wise classification will improve the reliability of the M-FISH imaging technique in identifying subtle and cryptic chromosomal abnormalities for cancer diagnosis and genetic disorder research.

Copyright © 2006 Y.-P. Wang and A. K. Dandpat. This is an open access article distributed under the Creative Commons Attribution License, which permits unrestricted use, distribution, and reproduction in any medium, provided the original work is properly cited.

### 1. INTRODUCTION

The whole human genome information is contained in the 24 classes of chromosomes. Multicolor fluorescence in situ hybridization (M-FISH) imaging is a recently developed molecular cytogenetic technique for rapid visualization of genomic aberrations at the chromosomal level [1–3]. Cancers and genetic diseases can be identified by analyzing chromosomal rearrangements such as inversions, deletions, and translocations. The M-FISH imaging approach overcomes the resolution limitation of the conventional chromosome banding technique when analyzing and interpreting complex and cryptic chromosomal abnormalities. Therefore, it has found widespread applications such as the prenatal and postnatal diagnosis, the identification of gene amplifications and deletions in tumors, and the detection of genetic markers and subtelomeric rearrangements in idiopathic mental retardation [3].

The M-FISH imaging technique is based on the simultaneous hybridization of a 24-chromosome specific probe pool [1, 3, 4]. By simultaneously viewing the multiple-labeled cell specimens in different color channels, it is possible to distinguish each human chromosome by means of the pixel-wise classification. Figure 1 shows that the 22 autosomes and the two sex chromosomes (the right panel) are displayed with 24 pseudocolors with the pixel-wise classification from a set of 6-channel FISH images (the left panel). The M-FISH images (shown on the left panel of Figure 1) are captured with different wavelengths using a microscope equipped with a filter wheel after the chromosomes are stained with 6 dyes. The intensity of the image at each spectral channel represents the level of binding for each probe. The M-FISH imaging technique is also called the color karyotyping, by which chromosomal abnormalities can be rapidly visualized. For a normal cell, all the pixels in each chromosome should be represented with one identical

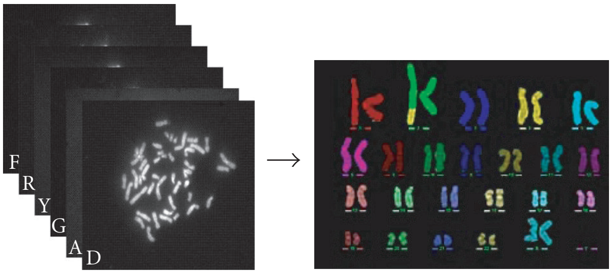

Figure 1: An illustration of the color karyotyping. The 24 classes of chromosomes are classified from the six-channel spectral image data sets (left panel); each class is displayed in a different pseudocolor (right panel). In the six-channel M-FISH image set (left figure), chromosome images are labeled with dyes DAPI (D), S Aqua (A), Red (R), S Green (G), S Red (Y), and S Gold (F), which can be captured using a microscope equipped with a filter wheel. The DAPI is used as a counter stain to visualize all the chromosomes.

pseudocolor. For a cancerous cell, however, different colors might show up in a chromosome as a result of the chromosomal rearrangements or the exchange of DNA materials between chromosomes. Therefore, by analyzing the color karyotype, geneticists can easily determine if any of the genetic material on the chromosomes has been lost or rearranged, and use it for the study of cancers and genetic disorders.

As a whole genome staining technique, M-FISH imaging promises a rapid and high-resolution genetic diagnosis with the help of automated computer image analysis [4–7]. The reliability of this molecular diagnosis technique, however, has not reached the level for clinical use [8]. The technique largely depends on the accuracy of pixel-wise classification from the multichannel FISH imaging data. Even for a normal cell, the classification accuracy can not be 100% correct. This will become especially challenging when applying the technique to cancerous cells; it is difficult to determine if the color change in a chromosome is due to the classification error or due to the chromosomal anomalies. Therefore, a crucial step is to improve the pixel-wise classification accuracy.

Two major problems that could affect the accuracy of pixel-wise classification are (1) *the data normalization* and (2) *the design of classifiers*. For the M-FISH images, the redundancy and misalignment between the multiple spectral channels are the primary factors that cause the subsequent classification to be less accurate [9]. Therefore, data normalization approaches such as the registration and dimension reduction must be performed before the classification. In the paper, we introduce two novel approaches for multichannel image registration and feature selection. Currently the Bayesian classifier has been used and implemented in commercial software packages [9, 10]. This model assumes that each class of chromosomes follows a Gaussian normal distribution in the feature space, which might not be realistic. We introduce a more accurate model based on the fuzzy clustering approaches [11]. The whole procedure combines wavelet-based normalization with fuzzy clustering, which is outlined in Figure 2. The proposed algorithm takes into account the intrinsic relationship between the feature selection and the classifier design.

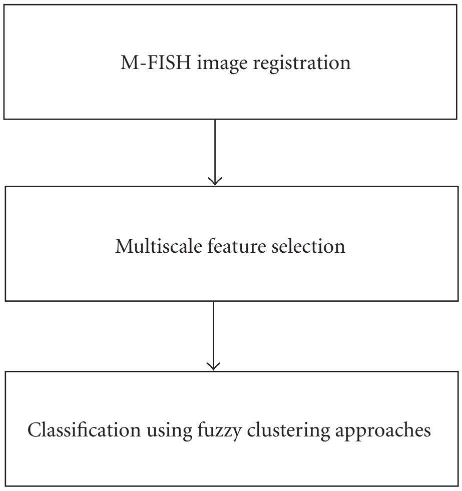

Figure 2: The outline of the proposed M-FISH classification procedure.

The rest of the paper is organized as follows. Section 2 introduces a multiresolution registration algorithm that can improve both the computational speed and the accuracy. Section 3 presents an approach for feature selection, which combines the principal component analysis with a shift-invariant wavelet representation. Section 4 describes fuzzy clustering approaches and compares them with the currently used Bayesian classifier [10]. Section 5 evaluates the proposed algorithms on a real M-FISH dataset [12] that we have established. Section 6 concludes the paper with a discussion on the advantages of the proposed approaches and their impact on the diagnosis of cancers and genetic disorders.

### 2. MULTIRESOLUTION M-FISH IMAGE REGISTRATION

### 2.1. M-FISH image registration problem

In an M-FISH image set, each pixel for a particular chromosome is represented by the six-channel spectral data. The representation can be denoted by

$$\mathbb{X}^n = (x_1^n, x_2^n, \dots, x_c^n)^t, \quad n = 1, 2, \dots, N, \tag{1}$$

where $N$ is the number of pixels in the image and $c$ is the number of spectral channels (here $c = 6$). The spectral representation of (1) is used as features for a classifier to perform the pixel-by-pixel classification.

The pixel misalignment between different spectral channels is a serious problem that could result in lower classification rate. The problem is caused by the mechanical vibrations in the filter wheel, axial and lateral chromatic aberrations, microscope set-up, and so forth, For a detailed analysis of these causes, see [10]. The registration technique seeks optimal geometric transformation $T$ between the two-channel spectral images $A$ and $B$. More specifically, the transformation $T$ is determined by minimizing an objective function $F(I_A(a), I_B(T(a)))$:

$$T' = \operatorname{Arg}\min_{T} F(I_A(a), I_B(T(a))), \tag{2}$$

where the objective function $F$ measures the similarity between the two images $A$ and $B$, which have the intensity of $I_A(a)$ and $I_B(b)$, respectively.

The selection of similarity criteria will be discussed in the appendix. In the paper, we have found that the mutual information (MI) criterion is extremely suitable for M-FISH image registration. The registration with MI criterion is based on the maximization of the statistical dependence of pixel intensities in the reference and registered images. It does not directly depend on image intensities. Therefore, it is very suitable for multispectral image registration such as the M-FISH data; the image of each channel differs in the intensity but exhibits statistical correlation. The experiments in Section 5 will confirm such observation.

The geometric transformation $T$ can be (1) a translation of pixels in horizontal or vertical directions; (2) a rotation about certain axis; (3) a scaling; (4) a horizontal or vertical shear, and/or a combination of any of the above. The transformations consisting of (1), (2), and (3) are called the rigid body transformation, by which every pixel in the two images can be mapped by linear equations. The nonrigid transformation is more complex; the determination of such transformation usually involves the identification of landmark points [13]. In the paper, we assume the affine transformation between the two channel images. Specifically, the geometric transformation is characterized by the following equations:

$$\begin{aligned} x_2 &= t_x + s x_1 \cos(\theta) - s y_1 \sin(\theta), \\ y_2 &= t_y + s x_1 \sin(\theta) + s y_1 \cos(\theta), \end{aligned} \tag{3}$$

where $x_2$, $y_2$ are the coordinates of the image to be registered; $x_1$, $y_1$ are the coordinates of the reference image; $t_x$, $t_y$ are the translation parameters; $s$ is the scaling parameter, and $\theta$ is the angle of rotation. The DAPI channel image is usually taken to be the reference image because all the chromosomes in the spread cells are stained in this image.

### *2.2. Multiresolution optimization*

The minimization of objection function in (2) is a multidimensional nonlinear optimization problem. There are several numerical optimization methods that can be chosen,

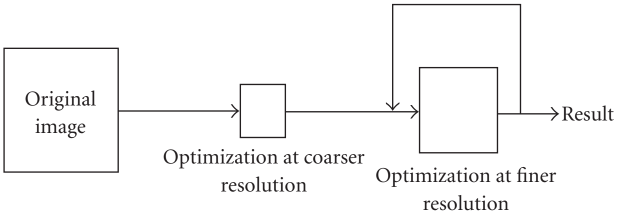

Figure 3: The multiresolution registration scheme where orthogonal and biorthogonal wavelet transforms were used.

which often involve the computation of gradients or derivatives. We have used the Powell direction set algorithm [14]. It is a multidimensional optimization method consisting of several one-dimensional minimizations. The objection function $F(P)$ of $n$ variables is minimized using an iterative procedure. The algorithm starts with a point "$P$" in an "$n$" dimensional space and proceeds to the next point by the line minimization approaches such as the Brent method. The procedure continues until a set of "$n$" linearly independent and mutually conjugate directions are found. In the ideal case, the algorithm is expected to find the global minima [14]. However, this algorithm sometimes converges in the local minima.

We introduce a multiresolution approach to overcome such difficulty. The idea of performing registration in a multiresolution framework has been proposed in other works [15, 16]. Figure 3 shows the diagram of the procedure. We first perform a multiresolution decomposition of the image such that the numerical optimization can be performed at the coarsest resolution, which can significantly reduce computational time. The parameters found at the coarse resolution are then used as initial values for the optimization algorithm at the next higher resolution level. This procedure is repeated until it reaches the original image resolution. There are several advantages to the proposed multiresolution technique. First, it is computationally effective. The optimization performed at coarse resolution can give a better estimation of initial values as opposed to the random selection when implementing the Powell algorithm. This will accelerate the convergence of the iteration, thus reducing computational time. Second, the algorithm is more robust to noise. The multiresolution algorithm finds near-optimal solution at the coarse resolution, thereby reducing the risk of trapping into false local minima. Section 5.2 will compare the image registration with and without multiresolution approach and the effect of initial values on the registration accuracy. After the transformation parameters are found, we perform a simple linear spline interpolation for geometric correction.

When implementing multiresolution decomposition, we used the conventional wavelet transform, which is different from the transform used for feature selection in Section 3.2. The decomposition uses a pyramid algorithm; from the coarse resolution to fine resolution the decomposition coefficients are downsampled by a factor of 2. We have used the orthogonal Haar and biorthogonal spline wavelets [17]. The effect of these wavelet filters on the registration accuracy will be evaluated in Section 5.2.

### 3. DIMENSION ANALYSIS IN THE MULTIRESOLUTION DOMAIN

### 3.1. Feature space and dimension reduction

The 6-dimensional vectors in (1) are used as features to classify the pixel into one of the 24 classes of chromosomes. More specifically, the 24 classes of chromosomes form 24 clusters in the 6-dimensional feature space. Ideally, these clusters should be well separated. However, due to the overlap of emission spectra and hybridization noises, redundancies exist between multiple channels. For efficient classification, it is advantageous to compress the high-dimensional feature space into the low-dimensional representation such that irrelevant information can be reduced. One of the most commonly used methods is the principal component analysis (PCA) or the eigenvalue analysis. In addition to its popular use in face recognition [18, 19], the eigenvalue-based approach has been applied for DNA microarray analysis to filter out the "eigengene" that is inferred to represent noises or experimental artifacts [20]. In order to extract the primary representations of the data, the PCA is in fact seeking the directions along which the dispersion or variance of the data cloud is maximal.

For the feature vector (1), the $c \times c$ covariance matrix is calculated as

$$\operatorname{Cov}(\mathbb{X}^n) = \mathcal{E}(\mathbb{X}^n - \mathcal{E}(\mathbb{X}^n))(\mathbb{X}^n - \mathcal{E}(\mathbb{X}^n))^t, \quad (4)$$

where $\mathcal{E}(\mathbb{X}^n)$ is the mean of $\mathbb{X}^n$. The linear transform that maximizes the data covariance turns out to be the eigenvectors, which are derived by the following eigendecomposition:

$$\operatorname{Cov}(\mathbb{X}^n) = UFU^t, \qquad F = \operatorname{diag}(\lambda_1, \lambda_2, \ldots, \lambda_c), \quad (5)$$

where $U$ is the eigenvector matrix and $F$ is the diagonal matrix consisting of eigenvalues that are sorted in decreasing order.

The dimension reduction can be obtained by discarding the $l$ least significant eigenvectors and then applying the following linear transform:

$$\mathbb{Y} = U_l(\mathbb{X}^n - \mathcal{E}(\mathbb{X}^n)), \quad (6)$$

where $U_l$ is the submatrix of $U$ by discarding its last $l$ rows. The mean square error of such approximation or dimension reduction is given by

$$\text{MSE} = \sum_{k=l}^{c} \lambda_k. \quad (7)$$

The ratio

$$R_l = \frac{\sum_{k=1}^{c-l} \lambda_k}{\sum_{k=1}^{c} \lambda_k} \quad (8)$$

indicates the relative significance of the remaining $c - l$ channel expression levels over the overall expression levels.

In the paper, instead of performing the PCA directly on the M-FISH images, we introduce a novel approach to perform the PCA in a multiresolution domain. This approach can offer adaptive reduction of redundancies. The idea is that the spectral data is decomposed into low- and high-frequency components with wavelet transform such that different levels of dimension compression can be performed according to the corresponding degrees of redundancy. At high resolution the wavelet coefficients contain the high-frequency components of the M-FISH image, which usually correspond to noises or redundancies. Therefore, we can set more eigenvectors to be zeros. On the other hand, at the coarse resolution we can discard less eigenvectors because there are little redundancies. This scheme will result in adaptive data smoothing or compression of the feature space.

The PCA analysis is in fact the correlation analysis. In order to be able to apply the PCA in the wavelet domain, a nondecimated wavelet transform is more appropriate. The cross-channel data correlation analysis depends greatly on the well-alignment of feature positions between different spectral channels. The translation-invariant or nondecimated transforms can facilitate such analysis. With such transforms, if a spatial correlation exists between the different channel images, it will be maintained in the wavelet domain. In the next section, we will review a family of differential spline wavelet representations [21, 22] that we have designed. These nondecimated representations have both shift-invariant property and computational simplicity. The combination of these representations with the PCA offers adaptive reduction of the redundancy.

### 3.2. Review of translation-invariant wavelet representations

The translation-invariant wavelet representations [21] designed by the first author are the generalizations of the wavelet frames proposed in [23]. They have been successfully used for chromosome image enhancement [24]. These wavelets are taken as the first- and second-order derivatives of the spline functions:

$$\psi^n(x) = \frac{d}{dx}\beta^{n+1}(x) \quad \text{or} \quad \psi^n(x) = \frac{d^2}{dx^2}\beta^{n+2}(x), \quad (9)$$

where $\beta^n(x)$ is the $B$-spline of order $n$.

If we define the smoothing and wavelet transforms of a signal $f$ at the dyadic scales as $S_{2^j} f$ and $W_{2^j} f$, we can compute the smoothing operation and wavelet transforms using a fast iterative algorithm:

$$\begin{gathered} S_{2^j} f = S_{2^{j-1}} f * h_{\uparrow 2^{j-1}}, \\ W_{2^j} f = S_{2^{j-1}} f * g_{\uparrow 2^{j-1}}, \quad j = 1, 2, \ldots, J, \end{gathered} \quad (10)$$

where $\{h\}$ and $\{g\}$ are the lowpass and highpass filters; and $\uparrow 2^j$ is the up-sampling operation. Conversely, the signal $f$ can be recovered by

$$S_{2^{j-1}} f = S_{2^j} f * h_{\uparrow 2^{j-1}} + W_{2^j} f * \tilde{g}_{\uparrow 2^{j-1}}. \quad (11)$$

Table 1: FIR filters for decomposition and reconstruction based on the 0th-order spline.

| Taps | $h$ | $\tilde{h}$ | $g$ | $\tilde{g}$ |
|---|---|---|---|---|
| −1 | 1/2 | 1/2 | −1 | 1/4 |
| 0 | 1/2 | 1/2 | 1 | 1/4 |

Table 2: FIR filters for decomposition and reconstruction based on the 1st-order spline.

| Taps | $h$ | $\tilde{h}$ | $g$ | $\tilde{g}$ |
|---|---|---|---|---|
| −2 | — | — | — | 1/16 |
| −1 | 1/4 | 1/4 | −1 | 5/16 |
| 0 | 1/2 | 1/2 | 1 | −5/16 |
| 1 | 1/4 | 1/4 | — | 1/16 |

This iterative algorithm is called the pyramid-like algorithm [21], which is similar to the conventional pyramid algorithm [17] except that no downsampling or decimation is performed.

The above wavelet transforms are translation-invariant [21]. This property is ideal for analyzing correlations between the multispectral channel images. When the M-FISH images are decomposed into the multiresolution domain, the authentic signal patterns maintain strong cross-scale correlations while noncorrelated noise components are mainly left out at the high resolution. The other advantage of the decomposition (10) is that the filters $\{h\}$ and $\{g\}$ are binomials and difference operations; only additions are needed when they are implemented. Filters of any orders can be found in [21]. We list several filters of lower orders that were used in our experiments.

*The Haar-like wavelets*

In the extreme case, when the order of the spline is taken as 0 we obtain the Haar-like wavelets. Table 1 lists the finite impulse responses (FIRs) of the decomposition and reconstruction filters. These filters (except the normalization constant) are identical to the conventional Haar filters for orthogonal wavelet transforms. The difference between them is that no downsampling is performed in the decomposition formula (10).

*The linear and cubic differential wavelets*

We list the FIRs of linear and cubic differential spline wavelets, which have been used to evaluate the proposed algorithms in the work. Tables 2 and 3 list the FIRs for these filters, which are derived from the linear and cubic $B$-splines. Different orders of spline filters will have different smoothing effects.

Table 3: FIR filters for decomposition and reconstruction based on the cubic spline.

| Taps | $h$ | $\tilde{h}$ | $g$ | $\tilde{g}$ |
|---|---|---|---|---|
| −4 | — | — | — | 1/256 |
| −3 | — | — | — | 9/256 |
| −2 | 1/16 | 1/16 | — | 37/256 |
| −1 | 1/4 | 1/4 | −1 | 93/256 |
| 0 | 3/8 | 3/8 | 1 | −93/256 |
| 1 | 1/4 | 1/4 | — | −37/256 |
| 2 | 1/16 | 1/16 | — | −9/256 |
| 3 | — | — | — | −1/256 |

### 3.3. Dimension analysis in the shift-invariant wavelet domain

The procedures of dimension analysis or feature selection in the shift-invariant multiresolution domain are detailed as follows.

(1) For each pixel $\mathbb{X}^n$, $1 \le n \le N$, in (1) (for simplicity, we omit the index $n$ in the following), we first perform the multiscale wavelet decompositions of each channel image $x_l$, $l = 1, 2, \dots, 6$, using (10) to have shift-invariant multiscale representations $\{\mathbb{S}^J x_l,\ W^j x_l,\ j = 1, 2, \dots, J\}$.

(2) Construct a new feature vector consisting of multiresolution wavelet decomposition components of $\mathbb{X}^n$ at each resolution, $\widetilde{\mathbb{X}}_j = [W_j x_1, W_j x_2, \dots, W_j x_6]$, $j = 1, 2, \dots, J$. Similar procedure is conducted with the low-frequency components $\widetilde{\mathbb{S}}_J = [S_J x_1, S_J x_2, \dots, S_J x_6]$.

(3) Perform the PCA-based dimension reduction or feature selection at each scale using (6):

$$\begin{aligned} \widetilde{W}_j \mathbb{X} &= (U_j^1)\left(\widetilde{\mathbb{X}}_j - \mathcal{E}(\widetilde{\mathbb{X}}_j)\right), \quad 1 \le j \le J, \\ \widetilde{\mathbb{S}}_J \mathbb{X} &= (U_J^2)\left(\widetilde{\mathbb{S}}_J - \mathcal{E}(\widetilde{\mathbb{S}}_J)\right), \end{aligned} \tag{12}$$

where $U_j^1$ and $U_J^2$ represent the sub-eigenmatrix of $\widetilde{\mathbb{X}}_j$ and $\widetilde{\mathbb{S}}_J$, respectively. Here, the eigenvalues or eigenvectors at different resolutions are selected adaptively in terms of the degree of correlations. More specifically, at finer resolution more eigenvectors are set to be zeros while at coarser resolution more eigenvectors are kept.

(4) Reconstruct each spectral component $x_l$, $1 \le l \le 6$, of $\mathbb{X}^n$ using the wavelet reconstruction formula of (11).

The advantage of the above algorithm is that the feature selection or dimension analysis can be performed in an adaptive way. In performing the PCA, one difficulty is how to select the number of principal components. This can be solved by using multiresolution scheme. When performing PCA in the multiresolution domain, at high resolutions the majority of the eigen-components correspond to noise or artifact. Therefore, we can set more eigenvectors or eigenvalues to be zeros at high resolutions. On the other hand, we can keep more eigenvalues at coarse resolutions because noise is smoothed out. This multiresolution scheme enables us to obtain an adaptive feature selection in terms of level of redundancy in the M-FISH images. The advantage of such method will be demonstrated with real examples in Section 5.

### 4. FUZZY CLUSTERING APPROACHES FOR M-FISH IMAGE CLASSIFICATION

Since the introduction of M-FISH imaging, several pixel-wise classification approaches have been studied. Among them, Bayesian classifier is widely used and implemented in commercial software packages [9, 10, 25]. The Bayesian model assumes that each class of chromosomes follows a Gaussian normal distribution in the feature space; each cluster is determined by the mean and the variance of the Gaussian probability density function. Because of the spectral overlap, this assumption is not realistic. We introduce the fuzzy clustering approaches, which consider the fact that the clusters are not completely well separated and each pixel is assigned to a cluster by the degree between 0 and 1. In this section, we review the fuzzy clustering approach. The advantage of these fuzzy classifiers over the Bayesian classifier on real M-FISH images will be demonstrated in Section 5.

### 4.1. *Fuzzy-clustering-based classification*

Clustering is a technique to divide a multidimensional data set into clusters or classes of similar attributes. The traditional k-means or hard c-means clustering is obtained by minimizing a dissimilarity (or distance) function given by

$$J = \sum_{i=1}^{c} J_i = \sum_{i=1}^{c} \sum_{x_k \in G_i} d_{ki}, \tag{13}$$

where $c_i$ is the centroid of cluster $i$ and $d_{ki} = d(x_k, c_i)$ is the distance between the $i$th centroid $c_i$ and the $k$th data point $x_k$. Typically, the Euclidean distance $\|x_k - c_i\|^2$ is used as the dissimilarity measure.

The minimization according to (13) leads to the k-means clustering algorithm or the hard c-means (HCM) clustering. The feature space is partitioned into groups, which can be defined by a $c \times N$ binary membership matrix $U$:

$$\mu_{ij} = \begin{cases} 1 & \text{if } \|x_j - c_i\|^2 \le \|x_j - c_k\|^2 \text{ for each } k \ne i, \\ 0 & \text{otherwise,} \end{cases} \tag{14}$$

where the element $u_{ij}$ is 1 if the $j$th data point $x_j$ belongs to the group $i$, and 0 otherwise.

With the HCM-based approach each data sample is assigned to exactly one cluster. Thus we obtain a crisp partitioning with sharp boundaries between the clusters. The fuzzy c-means clustering (FCM) is an improvement over the HCM by employing a fuzzy partitioning such that the sample point can belong to all groups with different membership grades between 0 and 1. The dissimilarity function used in the FCM is given by

$$J(U, C_1, C_2, \ldots, C_c) = \sum_{i=1}^{c} J_i = \sum_{i=1}^{c} \sum_{j=1}^{N} u_{ij}^m d_{ij}^2, \tag{15}$$

where $u_{ij}$ is between 0 and 1; $c_i$ is the centroid of cluster $i$; and $d_{ij}$ is the Euclidean distance between the $i$th centroid $c_i$ and the $j$th point. $m$ is the fuzzifier that controls the degree of fuzziness; higher values make the boundary between the clusters softer while lower values make them harder. Moreover, the sum of membership values should be one, that is, $\sum_{i=1}^{c} \sum_{j=1}^{N} u_{ij} = 1$.

If additional terms are introduced to regularize the above minimization problem such that

$$J(U, C_1, C_2, \ldots, C_c) = \sum_{i=1}^{c} \sum_{j=1}^{N} u_{ij}^m d_{ij}^2 + \sum_{i=1}^{c} w_i \sum_{j=1}^{N} (1 - u_{ij})^m, \tag{16}$$

one can obtain the possibilistic c-means clustering (PCM).

The class of chromosomes to which the pixel $j$ belongs is determined by the maximum value of the membership function $u_{ij}$. The objective functions in (13), (15), and (16) are usually minimized by an alternative two-step numerical optimization algorithm [11]. For the FCM, the iteration equations are

$$c_i = \frac{\sum_{j=1}^{N} u_{ij}^m x_j}{\sum_{j=1}^{N} u_{ij}^m}, \qquad u_{ij} = \frac{1}{\sum_{k=1}^{c} \left[ d_{ij}/d_{ik} \right]^{2/(m-1)}}. \tag{17}$$

For the PCM, the update equations are

$$c_i = \frac{\sum_{j\ne 1}^{N} u_{ij}^m x_j}{\sum_{j=1}^{N} u_{ij}^m}, \qquad u_{ij} = \frac{1}{1 + \left[ d_{ij}^2/w_i \right]^{1/(m-1)}}. \tag{18}$$

The membership $u_{ij}$ and the cluster centroids $c_i$ are updated in an alternating way according to the above equations until the change of membership degrees is less than a predefined threshold [11].

### 5. EXPERIMENTAL RESULTS

This section evaluates the performance of the proposed algorithms when applied to the real M-FISH image data sets. For the registration algorithm, the similarity metrics and the selection of initial values and wavelet filters could result in different accuracy. Therefore, we also simulated an image with known image transformation to evaluate the effects of these parameters.

### 5.1. *M-FISH database*

We have collaborated with Advanced Digital Imaging Research (ADIR), LLC, for this research. A database consisting of 200 M-FISH-labeled human chromosome spread images has been established by ADIR [12]. The database contains six-channel image sets recorded at different wavelengths. The specimens were prepared with probe sets from Applied Spectral Imaging (ASI), ADIR, Cytocell, and Vysis. The database contains 200 spreads from 33 slides from five different laboratories. The specimens include 74 normal male, 8 normal

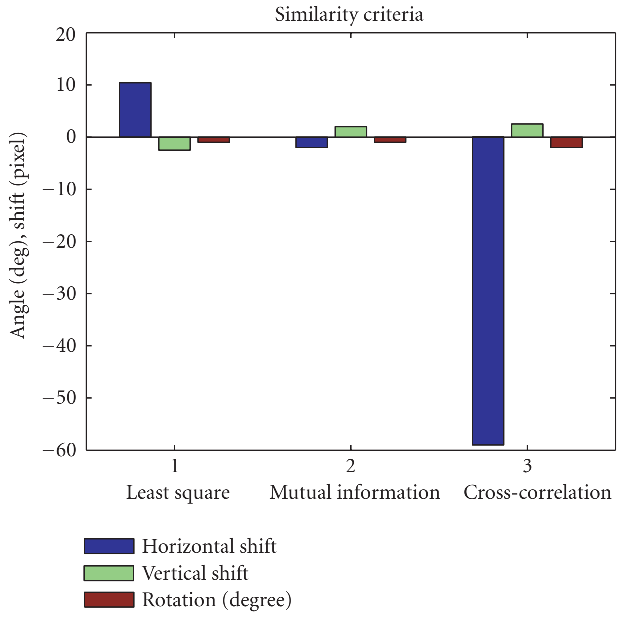

Figure 4: A comparison of registration accuracies when using different similarity metrics. The mutual information criterion turns out to be the best.

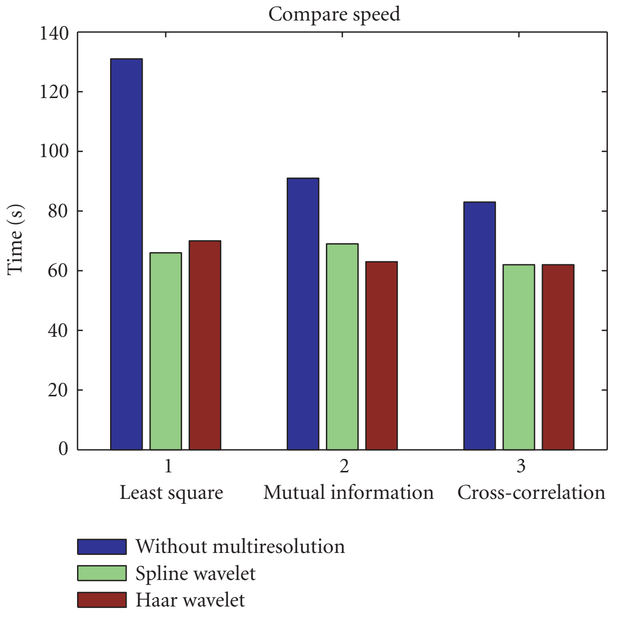

Figure 5: A comparison of computational speeds with and without multiresolution approach under different similarity metrics.

female, 99 abnormal spreads, and 17 more that are of low specimen quality. There are 50 different chromosomal aberrations represented, including both numerical abnormalities and structural rearrangements. Chromosome spread quality ranges from excellent to very difficult. Each data set consists of six-channel images labeled with different dyes. For validation purposes, a classification map is also included, which was established by experienced cytogeneticists. The classification map is stored as an image file and the gray level of each pixel represents the class to which it belongs. The background pixels are labeled 0, and the pixels in a region of overlap are labeled $-1$. This data file serves as the ground truth to evaluate the accuracy of M-FISH image classification algorithms. This comprehensive image database is a valuable source for M-FISH imaging studies.

### *5.2. Evaluation on multiresolution image registration*

The performance of multiresolution registration algorithms depends on a few factors such as the selection of similarity metrics. In order to evaluate the effect of these parameters, we have simulated an image with known geometric transformations. The simulated image was translated and rotated with known values, which were used as the ground truth when comparing with the computed transformation parameters using the proposed algorithms.

Figure 4 compares the classification accuracy of using different similarity metrics. The simulated image was shifted 2 pixels in the horizontal and vertical directions, and rotated 2 degrees. The $y$-axis of Figure 4 shows the computed transformation parameters. It turns out that the mutual information metric is the best among the three tested metrics.

In order to test if the multiresolution approach can reduce the computational time and improve the accuracy of registration, we tested the registration algorithm on the above simulated image with and without multiresolution scheme. We also tested the algorithm using different wavelet filters. Figure 5 compares the CPU time (in seconds). It indicates that multiresolution approach can significantly reduce the computational time, regardless of the similarity metrics used. A slightly faster speed can be obtained with Haar filters because of their shorter length. Figure 6 compares the accuracy with and without multiresolution scheme. It indicates that the multiresolution approach can also improve the accuracy of the registration. In the experiment, the nearly exact estimations were obtained with Haar and spline filters.

The numerical solution of (2) begins with the use of initial values. We have tested the effect of the selection of the initial values on both the computational speed and the accuracy of the registration. Figures 7 and 8 display the results of the computational speed and accuracy using different initial values and similarity metrics. The accuracy was computed as the relative error between the computed values and ground-truth values. It can be seen, although the initial values could affect the registration accuracy, the difference between them is not significant. This indicates the robustness of the proposed algorithm brought by the multiresolution search.

Finally, we tested the algorithm on the real M-FISH image database [12]. An objective way of evaluating the performance of image registration is to see if the subsequent pixel-wise classification can be improved. The five M-FISH image sets were selected to be representative, and they were from different focal planes and probes. Table 4 compares the accuracy of M-FISH image classification on five data sets

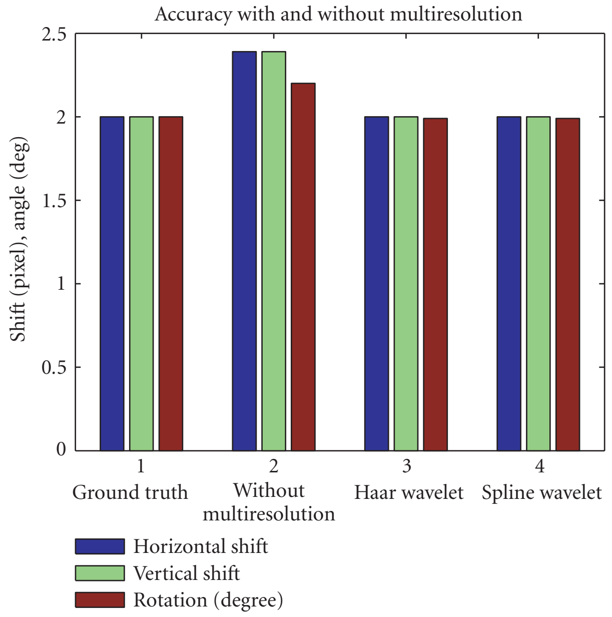

Figure 6: A comparison of registration accuracies with and without multiresolution approach. The values of geometric transformation parameters are displayed on $y$-axis.

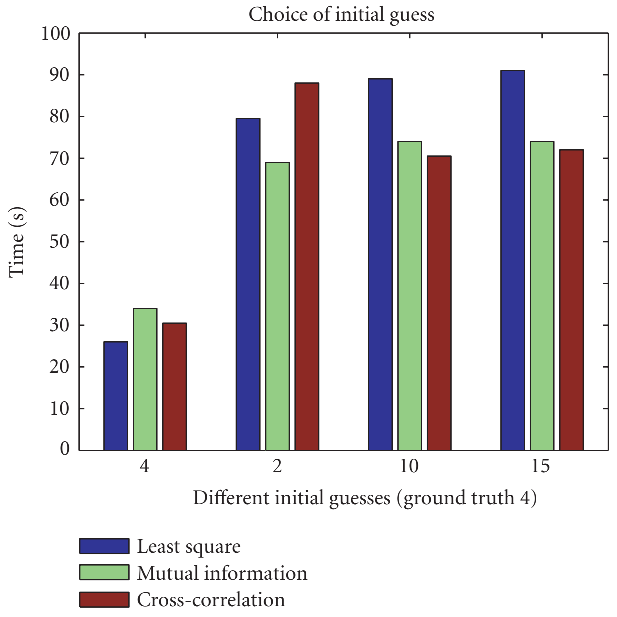

Figure 7: The computational speed is only slightly affected by the different choices of initial values, which indicates the robustness of the algorithm brought by the multiresolution scheme.

with and without registration, which demonstrates improved accuracy. The experiment confirms our observation that the colocalization of multispectral images can lead to a better classification accuracy. The images in the ADIR data set [12] have been well aligned using the commercial software, the result indicates that the proposed algorithm can still improve the registration accuracy. In case that the M-FISH images are not well registered, the proposed algorithm is expected to give more significant improvement.

### 5.3. Evaluation on the wavelet-based feature selection

In order to see if there is any redundancy between the M-FISH images, we have performed the dimension analysis on five sets of M-FISH images. We compared the classification accuracy using FCM with and without dimension reduction. The dimension analysis was performed on the six-channel spectral data using the PCA. We discarded the least significant eigenvector in the PCA transform described in Section 3.1 such that the dimension is reduced from 6 to 5. It can be seen from Table 5 that the dimension reduction or feature selection does lead to improved classification accuracy. This indicates that the reduction of redundancy between the multispectral FISH images is a necessary step that can increase the classification accuracy.

As discussed in Section 3, feature selection can be further improved by performing the PCA analysis in the shift-invariant wavelet domain. We have performed the multiscale PCA analysis using the algorithm of Section 3.3 on the same dataset as in Table 5. The results are listed in Table 6, which indicates that multiscale PCA can further improve the conventional PCA-based dimension analysis. In the

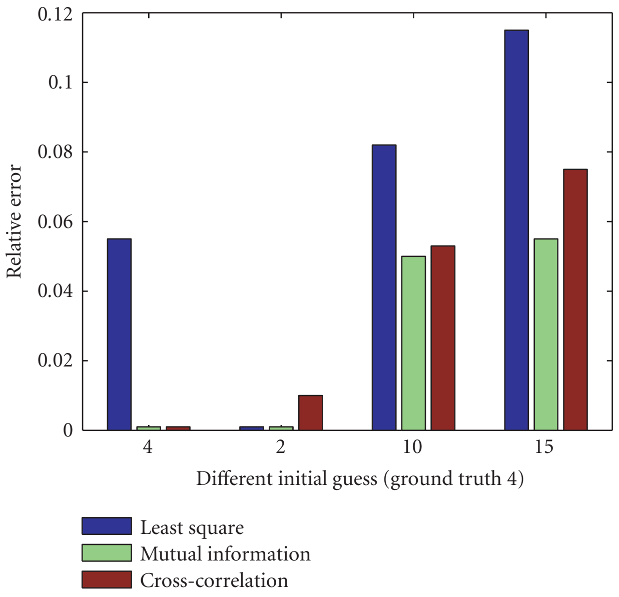

Figure 8: The effect of initial values on the registration accuracy. It indicates that the algorithm is insensitive to the choice of initial values because of the multiresolution algorithm. The $x$-axis is the index of different sets of initial values. The $y$-axis is the relative error.

experiment both the Haar-like and cubic spline wavelets were used, and the results are listed in the second and third row of the table. It can be seen that wavelet filters of different sizes produce comparable results. Because Haar-like filters have shortest sizes and take less time, we recommend the use of these filters in practice. Since the PCA transforms in the

Table 4: A comparison of classification accuracies (in percentage) by FCM with and without wavelet registration.

| Data set | 1 | 2 | 3 | 4 | 5 |
|---|---|---|---|---|---|
| No registration | 84.22 | 84.63 | 85.74 | 89.25 | 91.29 |
| Registration | 85.82 | 85.92 | 86.88 | 89.88 | 91.97 |

Table 5: A comparison of the classification accuracy (in percentage) with and without dimension reduction or feature selection, where FCM was used for the pixel-wise classification.

| Data set | 1 | 2 | 3 | 4 | 5 |
|---|---|---|---|---|---|
| Without dimension reduction | 72.13 | 82.31 | 80.23 | 85.27 | 89.91 |
| With dimension reduction | 79.03 | 83.13 | 82.33 | 87.19 | 90.22 |

Table 6: A comparison of classification accuracies (in percentage) using different wavelet filters, where FCM was used for the pixel-wise classification.

| Data set | 1 | 2 | 3 | 4 | 5 |
|---|---|---|---|---|---|
| Without wavelets | 72.13 | 82.31 | 80.23 | 85.27 | 89.91 |
| Haar-like wavelets | 85.27 | 83.44 | 84.69 | 90.44 | 90.66 |
| Cubic wavelets | 86.22 | 84.10 | 84.33 | 90.81 | 90.88 |

wavelet domain take more time than those in a single scale, two to three levels of wavelet decompositions are suggested.

### 5.4. A comparison of classification accuracy between different classifiers

We have compared different classifiers on a number of M-FISH data sets, and note that the Bayesian classifiers [10] were currently used in the commercial software. Because of the availability of the ground truth of the classification map for normal cells, the tests were conducted on images containing normal chromosomes. These ten images are representative, and are selected from different probes and focal planes. An extensive study on the clinical feasibility when analyzing cancerous cells will be conducted in the future. Table 7 lists the classification accuracies of testing 10 data sets using the Bayesian classifier, HCM, FCM, and PCM clustering. These M-FISH images have been registered. By comparing the classification accuracy, it can be seen that the FCM approach gives the highest average accuracy among the four classifiers tested. However, the PCM approach gives lowest covariance with a slight lower average accuracy than the FCM. The results indicate that fuzzy models including both the FCM and PCM provide more realistic models to classify chromosomes from the M-FISH imaging data.

### 6. CONCLUSIONS

The chromosome color karyotyping using automated computer image analysis can relieve the labor expensive and tedious procedure in a cytogenetic laboratory, thereby accelerating the use of this novel imaging technique for rapid identification of genomic aberrations. As cytogeneticists use M-FISH imaging to identify cryptic and complex chromosomal rearrangements, the increase of pixel-wise classification accuracy can be translated into improved diagnosis accuracy in identifying cancers and genetic diseases. Therefore, the accurate classification of chromosomes for normal cells is crucial towards the reliable use of the novel molecular diagnosis technique.

Table 7: A comparison of classification accuracies (in percentage) between different classifiers. The last row is the average. The FCM approach turns out to be the best in terms of the average accuracy.

| Data set | Bayes | HCM | FCM | PCM |
|---|---|---|---|---|
| 1 | 77.16 | 60.23 | 84.22 | 82.32 |
| 2 | 82.31 | 61.34 | 84.63 | 83.73 |
| 3 | 81.76 | 65.22 | 85.74 | 85.98 |
| 4 | 90.71 | 67.45 | 91.98 | 88.45 |
| 5 | 84.69 | 62.63 | 89.25 | 88.02 |
| 6 | 85.27 | 63.32 | 88.65 | 89.35 |
| 7 | 81.77 | 61.44 | 90.95 | 89.55 |
| 8 | 90.44 | 68.56 | 91.79 | 88.51 |
| 9 | 89.33 | 66.33 | 91.29 | 87.47 |
| 10 | 72.23 | 60.22 | 91.82 | 88.36 |
| Mean | 83.56 | 63.67 | 89.03 | 87.17 |
| Variance | 34.81 | 9.23 | 9.60 | 5.87 |

In the paper we focus on the pixel-wise classification of chromosomes from normal cells. This is an important step to implement before applying the technique to analyze abnormal chromosomes in a clinical laboratory. We have introduced a hybrid approach that combines fuzzy clustering with two innovative wavelet-based preprocessing techniques to improve the classification accuracy. The registration algorithm using a multiresolution approach improves both the computational speed and accuracy when finding geometric parameters for the correction of misalignment. The feature selection or dimensional analysis in the translation-invariant wavelet domain offers an adaptive reduction of redundancies that are inherent in the multispectral images. The features extracted from these two preprocessing approaches can substantially increase classification accuracy. Among several classifiers tested, fuzzy-clustering-based approaches including both FCM and PCM are more appropriate for classifying the M-FISH images because these more sophisticated models assume that a pixel can belong to more than one class. The testing of the proposed algorithms on the comprehensive M-FISH image data sets indicates that they can significantly improve the pixel-wise classification accuracy for normal cells, translating into improved reliability of this bioimaging technique. The evaluation of these approaches for clinical use needs further research.

### APPENDIX

### THE SIMILARITY METRICS

The similarity metrics measure how the pixels in the reference and registered images are mapped. Different choices of metrics will result in different accuracies of computing geometric transformation parameters [26]. We have evaluated the following three similarity metrics.

*The sum of squared differences*

This is defined as the sum of the squared differences of intensity values of the registered and reference images:

$$D(A,B) = \sqrt{\sum \left(I_A(a) - I_B(b)\right)^2}, \qquad (A.1)$$

where $I_A$ and $I_B$ are the intensities of reference and registered images, respectively. The coordinates of the two images are related by $b = T(a)$.

*The normalized cross-correlation*

This similarity measure is generally useful for small rigid or affine transformed images. For images $A$ and $B$, the normalized cross-correlation function is given by

$$\mathrm{Cor}(A,B) = \sum \frac{I_A(a)I_B(b)}{\sqrt{\sum I_A(a)^2}}. \qquad (A.2)$$

This metric is accurate in the case of white noise. However, it is not tolerant to local distortions.

*The mutual information*

This is based on the joint histogram between the reference and registered images. The mutual information is derived from information theory, which is related to the Kullback-Leibler distance between the probability density functions of the two images $A$ and $B$ [27]. More specifically, it is computed as

$$\begin{aligned} I(A,B) &= H(A) + H(B) - H(A,B) \\ &= \sum P_{AB}(a,b) \log \frac{P_{AB}(a,b)}{P_A(a)P_B(b)}, \end{aligned} \qquad (A.3)$$

where

$$\begin{aligned} H(A) &= -\sum P_A(a) \log P_A(a), \\ H(B) &= -\sum P_B(b) \log P_B(b) \end{aligned} \qquad (A.4)$$

are the entropies of images $A$ and $B$, respectively. $H(A,B)$ is the joint entropy between them:

$$H(A,B) = -\sum P_{AB}(a,b) \log P_{AB}(a,b), \qquad (A.5)$$

where $P_A$ and $P_B$ are the probability density functions or histograms of the images $A$ and $B$, respectively; and $P_{AB}$ is the 2D joint probability or joint histogram of the two images. When the two images are perfectly aligned, the mutual information is maximized [27].

### ACKNOWLEDGMENTS

The first author thanks his former colleagues, Drs. K. Castleman, F. Merchant, and Q. Wu of ADIR, LLC, for their help. This work has been supported by the NIH, University of Missouri Research Board, and the Faculty Research Grant.

### REFERENCES

[1] T. Ried, A. Baldini, T. C. Rand, and D. C. Ward, "Simultaneous visualization of seven different DNA probes by in situ hybridization using combinatorial fluorescence and digital imaging microscopy," *Proceedings of the National Academy of Sciences of the United States of America*, vol. 89, no. 4, pp. 1388–1392, 1992.

[2] E. Schröck, S. du Manoir, T. Veldman, et al., "Multicolor spectral karyotyping of human chromosomes," *Science*, vol. 273, no. 5274, pp. 494–497, 1996.

[3] M. R. Speicher, S. G. Ballard, and D. C. Ward, "Karyotyping human chromosomes by combinatorial multi-fluor FISH," *Nature Genetics*, vol. 12, no. 4, pp. 368–375, 1996.

[4] M. R. Speicher, S. G. Ballard, and D. C. Ward, "Computer image analysis of combinatorial multi-fluor FISH," *Bioimaging*, vol. 4, no. 2, pp. 52–64, 1996.

[5] P. M. Nederlof, S. van der Flier, J. Wiegant, et al., "Multiple fluorescence in situ hybridization," *Cytometry*, vol. 11, no. 1, pp. 126–131, 1990.

[6] R. Eils, S. Uhrig, K. Saracoglu, et al., "An optimized, fully automated system for fast and accurate identification of chromosomal rearrangements by multiplex-FISH (M-FISH)," *Cytogenetics and Cell Genetics*, vol. 82, no. 3-4, pp. 160–171, 1998.

[7] K. R. Castleman, T. P. Riopka, and Q. Wu, "FISH image analysis," *IEEE Engineering in Medicine and Biology Magazine*, vol. 15, no. 1, pp. 67–75, 1996.

[8] C. Lee, D. Gisselsson, C. Jin, et al., "Limitations of chromosome classification by multicolor karyotyping," *American Journal of Human Genetics*, vol. 68, no. 4, pp. 1043–1047, 2001.

[9] Y.-P. Wang, "M-FISH image registration and classification," in *Proceedings of 2nd IEEE International Symposium on Biomedical Imaging: Macro to Nano*, vol. 1, pp. 57–60, Arlington, Va, USA, April 2004.

[10] Y.-P. Wang and K. R. Castleman, "Normalization of multicolor fluorescence in situ hybridization (M-FISH) images for improving color karyotyping," *Cytometry Part A*, vol. 64, no. 2, pp. 101–109, 2005.

[11] M. A. Sutton, J. C. Bezdek, and T. C. Cahoon, "Image segmentation by fuzzy clustering: methods and issues," in *Handbook of Medical Imaging*, I. N. Bankman, Ed., pp. 87–106, Academic Press, San Diego, Calif, USA, 2000.

[12] "The ADIR M_FISH Image Database," http://www.adires.com/01/index.shtml.

[13] A. P. Dhawan, *Medical Image Analysis*, IEEE Press Series in Biomedical Engineering, chapter 9, John Wiley & Sons, New York, NY, USA, 2003.

[14] W. H. Press, S. A. Teukolsky, W. T. Vetterling, and B. P. Flannery, *Numerical Recipes in C: The Art of Scientific Computing*, Cambridge University Press, Cambridge, UK, 1988.

[15] A. A. Cole-Rhodes, K. L. Johnson, J. LeMoigne, and I. Zavorin, "Multiresolution registration of remote sensing imagery by optimization of mutual information using a stochastic gradient," *IEEE Transactions on Image Processing*, vol. 12, no. 12, pp. 1495–1511, 2003.

[16] J.-P. Djamdji, A. Bijaoui, and R. Maniere, "Geometrical registration of images: the multiresolution approach," *Photogrammetric Engineering & Remote Sensing*, vol. 59, no. 5, pp. 645–653, 1993.

[17] I. Daubechies, *Ten Lectures on Wavelets*, SIAM, Philadelphia, Pa, USA, 1992.

[18] M. Turk and A. Pentland, "Eigenfaces for recognition," *Journal of Cognitive Neuroscience*, vol. 3, no. 1, pp. 71–86, 1991.

[19] V. Brennan and J. Principe, "Face classification using a multiresolution principal component analysis," in *Proceedings of the IEEE Workshop on Neural Networks for Signal Processing (NNSP '98)*, pp. 506–515, Cambridge, UK, August-September 1998.

[20] O. Alter, P. O. Brown, and D. Botstein, "Singular value decomposition for genome-wide expression data processing and modeling," *Proceedings of the National Academy of Sciences of the United States of America*, vol. 97, no. 18, pp. 10101–10106, 2000.

[21] Y.-P. Wang, "Image representations using multiscale differential operators," *IEEE Transactions on Image Processing*, vol. 8, no. 12, pp. 1757–1771, 1999.

[22] Y.-P. Wang and S. L. Lee, "Scale-space derived from *B*-splines," *IEEE Transactions on Pattern Analysis and Machine Intelligence*, vol. 20, no. 10, pp. 1040–1055, 1998.

[23] S. Mallat and S. Zhong, "Characterization of signals from multiscale edges," *IEEE Transactions on Pattern Analysis and Machine Intelligence*, vol. 14, no. 7, pp. 710–732, 1992.

[24] Y.-P. Wang, Q. Wu, K. R. Castleman, and Z. Xiong, "Chromosome image enhancement using multiscale differential operators," *IEEE Transactions on Medical Imaging*, vol. 22, no. 5, pp. 685–693, 2003.

[25] B. Lerner, "Bayesian fluorescence in situ hybridisation signal classification," *Artificial Intelligence in Medicine*, vol. 30, no. 3, pp. 301–316, 2004.

[26] L. G. Brown, "A Survey of image registration techniques," *ACM Computing Surveys*, vol. 24, no. 4, pp. 325–376, 1992.

[27] P. Viola and W. M. Wells III, "Alignment by maximization of mutual information," in *Proceedings of IEEE International Conference on Computer Vision (ICCV '95)*, E. Grimson, S. Shafer, A. Blake, and K. Sugihara, Eds., pp. 16–23, IEEE Computer Society, Los Alamitos, Calif, USA, 1995.

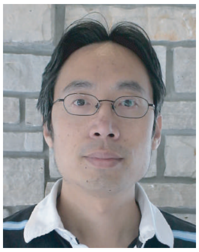

**Yu-Ping Wang** received the B.S. degree in applied mathematics from Tianjin University, China, in 1990, the M.S. degree in computational mathematics, and the Ph.D. degree in communications and electronic systems from Xi'an Jioatong University, China, in 1993 and 1996. After his graduation, he had visiting positions at the Center for Wavelets, Approximation and Information Processing, National University of Singapore and Washington University Medical School, St. Louis. From 2000 to 2003, he worked as a Senior Research Engineer at Perceptive Scientific Instruments, Inc, and then at Advanced Digital Imaging Research, LLC, Houston, Tex. In the fall of 2003, he returned to the academia as an Assistant Professor of Computer Science and Electrical Engineering at the University of Missouri-Kansas City. His current research interests lie in the interdisciplinary area of bioimaging, especially at the interface of wavelet research and genetic and genomic imaging.

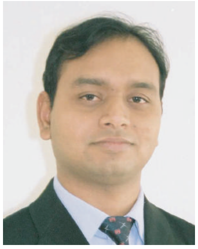

**Ashok Kumar Dandpat** received his B.Tech. degree in electrical engineering from Orissa University of Agriculture and Technology, India, in 2002, and his M.S. degree in electrical engineering from University of Missouri-Kansas City, in 2005. He was a Lecturer with the Department of Electrical Engineering, Padmanava College of Engineering, Orissa, India, and worked as a graduate Research Assistant in the area of biomedical imaging and wavelet analysis. Currently he is working as an Electrical Engineer in Specialty Group with the Black and Veatch Corporation, Kansas City, Kansas.

